# Hyper IgE Disorder Associated with Multiple Fused Primary Teeth: A Rare Clinical Occurrence

**DOI:** 10.5005/jp-journals-10005-1081i

**Published:** 2010-09-15

**Authors:** AJ Sai Sankar, MG Manoj Kumar, Y Samata, K Srikanth Reddy

**Affiliations:** 1 Professor, Department of Pedodontics and Preventive Dentistry, Sibar Institute of Dental Sciences, Guntur, Andhra Pradesh, India; 2 Professor and Head, Department of Pedodontics and Preventive Dentistry, Sibar Institute of Dental Sciences, Guntur Andhra Pradesh, India; 3 Senior Lecturer, Department of Oral Medicine and Radiology, Sibar Institute of Dental Sciences, Guntur, Andhra Pradesh, India; 4 Senior Lecturer, Department of Pedodontics and Preventive Dentistry, Sibar Institute of Dental Sciences, Guntur Andhra Pradesh, India

**Keywords:** Hyperimmunoglobulin, Pneumonia, Syndrome.

## Abstract

The hyperimmunoglobulin E syndrome (HIES) is a multisystem disorder that affects the dentition, skeleton, connective tissues and immune system. Little is known about oral manifestations of the syndrome. The purpose of this report was to describe a 6-year-old boy with suspected autosomal recessive HIES syndrome who had multiple fused primary teeth, which is a rare association with JOB syndrome. The patient gave a history of pneumonia and skin infections. Recognition of such case at an early age is necessary to reduce morbidity. As conclusion, treatment for this condition is life long administration of therapeutic doses of penicillinase-resistant penicillin, with the addition of other antibiotics or antifungal agents as required for specific infections.

## INTRODUCTION

Hyper-IgE syndrome (HIES), also called ‘Job syndrome’ and ‘hyper-IgE recurrent infection syndrome’ was first described as a primary immunodeficiency characterized by recurrent Staphylococcal skin abscesses, recurrent pneumonia with pneumatocele formation, eczema, pruritic dermatitis, eosinophilia and highly elevated levels of serum IgE. A coarse facial appearance, hyper extensibility of the joints, bone fractures, craniosytosis and prominent triangular mandible have been reported in many cases of HIES.^1^ The name Job’s syndrome was given by Davis in 1966 and Buckely subsequently related the condition to elevated levels of IgE.^2^

## CASE REPORT

**Fig. 1:**
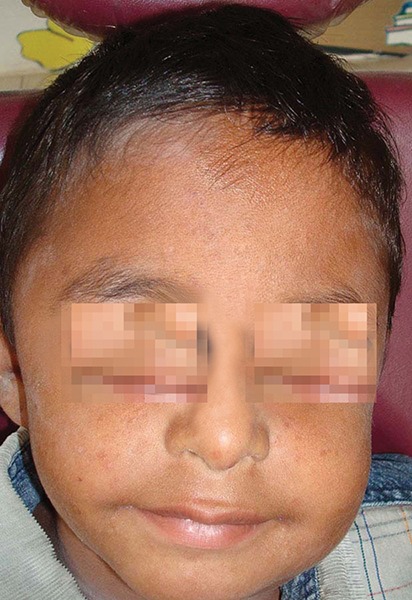
A 6-year-old male patient showing swelling on the left side of the face

**Fig. 2:**
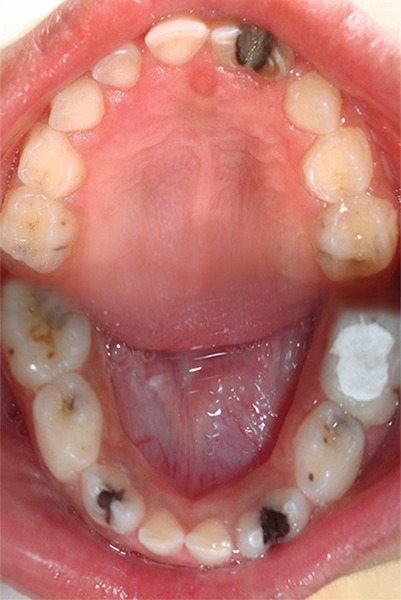
Maxillary and mandibular arches showing fused teeth irt 61, 62; 72, 73; 82, 83

**Fig. 3:**
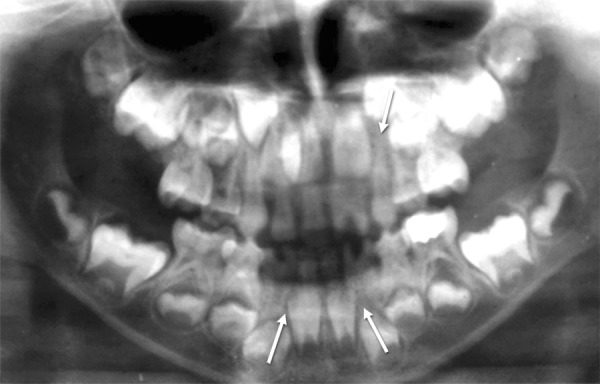
Orthopantamograph showing, missing permanent mandibular right and left lateral incisors and permanent maxillary left lateral incisor crypts

A 6-year-old male patient (Fig. 1) was referred to the department of pediatric dentistry, who was complaining of pain and swelling in relation to lower left back tooth region. Intraoral examination revealed advanced caries with pulpal involvement i.r.t 75, which was tender on vertical percussion. Also noticed, intraorally are fusion between mandibular right primary lateral incisor and canine, mandibular left primary lateral incisor and canine, as well as maxillary left primary lateral incisor and canine (Fig. 2). The clinical findings were confirmed radiographically, an OPG take showed congenitally missing 22, 32 and 42 in addition to the fused teeth (Fig. 3). Clinically the fused teeth had labial and lingual grooves and were also affected by deep dental caries. Moderate carious lesions were present i.r.t 55, 65, 84, 85, a deep carious lesion was present i.r.t 75. Past medical history revealed repeated hospitalization due to ill health of which twice was due to pneumonia. General physical examination showed skin rashes, scars due to previous skin abscesses, deep set eyes, prominent fore head and angular chelitis. The clinical picture suggested Hyper-IgE syndrome. The association of fused teeth with Hyper-IgE is being observed for the first time. To confirm the provisional diagnosis, the patient’s blood sample was sent for analysis and the report showed an elevated level of IgE.

**Fig. 4:**
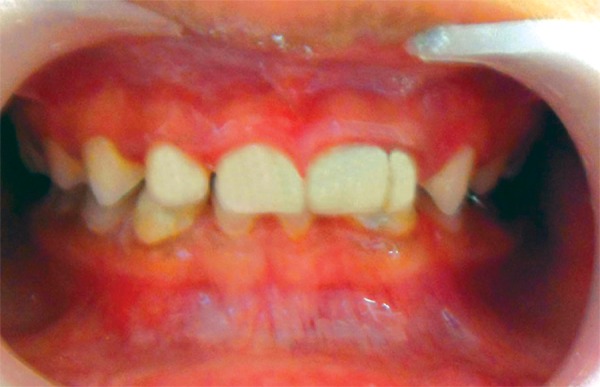
Fused teeth restored with composite resin and 75 restored with SSC

**Fig. 5:**
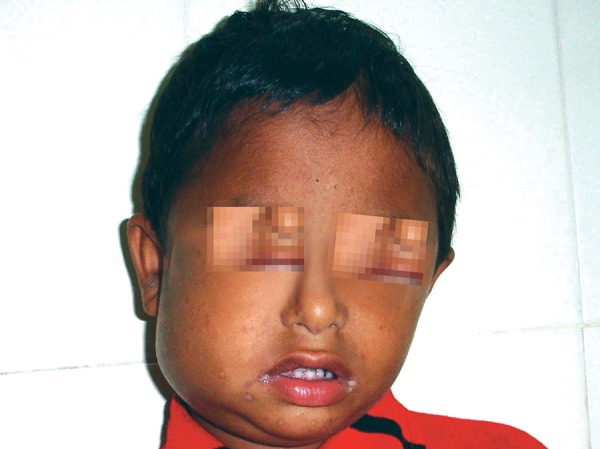
Patient showing angular chelitis, skin rashes and swelling in the right lower third of the face, on subsequent visit

**Fig. 6:**
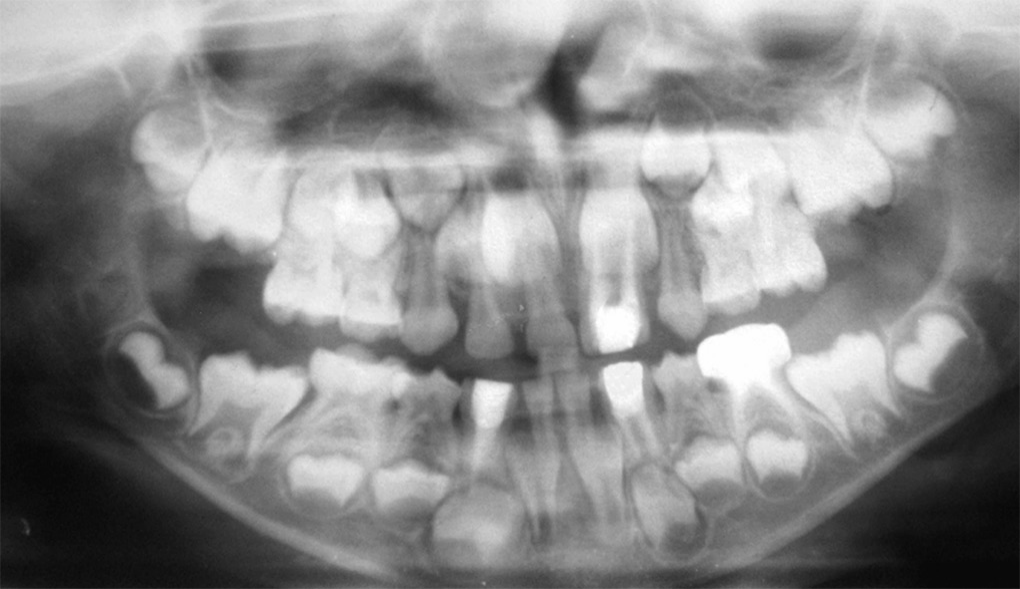
Follow-up OPG taken one year later revealed significant delay in resorption of deciduous teeth

This case was managed as follows. Emergency access opening of 75 was done in the first visit under antibiotic coverage to relive pain. In the later visits, the fused teeth were endodontically treated and restored with composite resin. 75 was restored with a stainless steel crown (Fig. 4) after pulpectomy whereas 55, 65, 84 and 85 were restored with glass ionomer cement. Then after 2 weeks he came back with swelling on the right side of the face (Fig. 5), which was previously restored with glass ionomer cement. An IOPA of the region was made which did not reveal any pathology then an ultrasound examination of the region was done and a diagnosis of right submandibular salivary gland abscess was made. An incision was made on the dependent part of the swelling and pus was drained. Pus was sent for culture which showed *Staphylococcus aureus* organism predominantly. The case was followed up for the next one year and an OPG was taken at the end of 1 year, which showed a significant delay in the developing dentition. Congenitally missing 22, 32 and 42 can be noticed (Fig. 6). None of the deciduous incisors show evidence of resorbtion nor have the permanent 1st molars erupted clinically.

## DISCUSSION

The Hyper-IgE syndrome is a multisystem disorder that affects the dentition, skeleton, connective tissue and the immune system.^1^ It is characterized by a triad of eczema, recurrent skin and lung infections, and a high serum level of IgE. The most consistent laboratory abnormalities in HIES are high serum IgE and eosinophilia. The serum IgE typically peaks at > 2000 IU/ml and may be elevated at birth. In the present case IgE level is 4102 I U/ml and eosinophil count is elevated at 5% suggesting HIES. WBC counts and IgA, IgM, IgG are usually normal, as noticed in the present case. Pneumonias typically present within the first few years of life. *Staphylococcus aureus* is the most common etiology with other pyogenic bacteria, such as *Streptococcus pneumoniae* and *Hemophilus influenzae* occurring frequently.^3^ In the present case, the patient was repeatedly hospitalized due to pneumonia.

Patients with this syndrome have a striking depression of acute inflammation, as evidenced by cold abscesses despite pronounced local infection, as seen in the present case, i.e. right submandibular salivary gland abscess. Abnormal neutrophil and monocyte chemotaxis have been documented. Lymphocyte function in HIES is abnormal. Delayed hypersensitivity responses to a variety of skin-test antigens are impaired in some, but not all patients. Mitogen-induced and antigen-induced lymphocyte transformation is impaired, especially with regard to Candida antigen and tetanus toxoid, and maybe related to the mucocutaneous candidiasis in many patients. Deficient suppressor T-cell numbers may account for increased IgE. Low or absent salivary and plasma anti-staphylococcal IgA likely contributes to the propensity to mucosal infections.^1^

Common facial appearance characterized by asymmetry, a fleshy nasal tip, deep-set eyes, and a prominent forehead: These are noticed in the present case. The skin of face typically has a coarse appearance with pronounced pores. Musculoskeletal abnormalities include scoliosis, osteopenia, minimal trauma fractures, degenerative joint disease, and craniosystosis.^3^

O’Connell et al reported that a disorder of tooth eruption is part of the hyper-IgE syndrome and the lack of resorption of the primary teeth may be associated with the persistence of Hertwig’s epithelial root sheath,^1^ supporting this is the significant delay in eruption and resorption of the dentition in the present case, as noticed on the OPG taken after 1 year of follow-up. Other known oral manifestations associated with HIES are periodontitis^4^ but this is the first case where HIES is associated with multiple fused teeth.

HIES diagnosis is based on a constellation of patients’ clinical and laboratory features, as no specific diagnostic test is available. A HIES scoring system developed at the national institute of health to phenotype patients with ADHIES is helpful.^4^

The genetic basis of the Hyper-IgE syndrome is unclear, as the most cases are sporadic. However, in many kindreds, autosomal dominant transmission, including male to male transmission has been reported.^1^ This child has clinical and laboratory features consistent with HIES. Having unaffected, consanguineous parents suggest the autosomal–recessive form of HIES in the present case.

Control and resolutions of the rash typically occur with anti-staphylococcal antibiotics or topical therapies, such as bathing in diluted bleach or swimming in chlorinated pools. Other modes of management include local debridment, surgical incision and drainage of infectious lesion.^3^

## CONCLUSION

Oral health maintenance and prompt treatment of dental infections are extremely important for patients with Hyper-IgE syndrome because significant morbidity can result from infections of odontogenic origin. Treatment for this condition is lifelong administration of therapeutic doses of penicillinase-resistant penicillin. In the addition, other antibiotics or antifungal agents are required for specific infections. The dentist plays an important role in recognizing the oral manifestations associated with the condition and the prompt treatment.
